# Sulforaphane ameliorates bisphenol A-induced hepatic lipid accumulation by inhibiting endoplasmic reticulum stress

**DOI:** 10.1038/s41598-023-28395-5

**Published:** 2023-01-20

**Authors:** Lixia Hong, Yide Xu, Dongdong Wang, Qi Zhang, Xiaoting Li, Chunfeng Xie, Jieshu Wu, Caiyun Zhong, Jinyan Fu, Shanshan Geng

**Affiliations:** 1 Department of Nutrition and Food Safety, Center for Global Health, School of Public Health, Nanjing Medical University, 101 Longmian Avenue, Jiangning District, Nanjing, 211166 China; 2 Department of Nutrition, Wuxi Maternal and Child Health Care Hospital, Wuxi, 214002 Jiangsu China

**Keywords:** Endocrine system and metabolic diseases, Cell biology, Metabolic disorders

## Abstract

The aim of the present study was to investigate the role of endoplasmic reticulum (ER) stress in bisphenol A (BPA) – induced hepatic lipid accumulation as well as the protective effects of Sulforaphane (SFN) in this process. Human hepatocyte cell line (LO2) and C57/BL6J mice were used to examine BPA-triggered hepatic lipid accumulation and the underlying mechanism. Hepatic lipid accumulation, triglycerides (TGs) levels, the expression levels of lipogenesis-related genes and proteins in the ER stress pathway were measured. It was revealed that BPA treatment increased the number of lipid droplets, the levels of TG and mRNAs expression of lipogenesis-related genes, and activated the ER stress pathway. These changes were inhibited by an ER stress inhibitor 4-phenylbutyric acid. SFN treatment abrogated BPA-altered hepatic lipid metabolism and ameliorated BPA-induced ER stress-related markers. Together, these findings suggested that BPA activated ER stress to promote hepatic lipid accumulation, and that SFN reversed those BPA effects by alleviating ER stress.

## Introduction

Bisphenol A (BPA), a commonly used component in various daily items, including plastic food containers and liners, is an environmental endocrine disruptor (EED)^1–3^. The major human exposure route to BPA include diet, water, and skin contact^4^. Our previous study has shown that BPA exposure causes peripheral insulin resistance, which is characterized by increased insulin and glucose levels^5^. Hepatic insulin resistance and hepatic steatosis are strongly interrelated^6^. Existing evidence suggests that type 2 diabetes mellitus is one of the major risk factors for non-alcoholic fatty liver disease (NAFLD) and may accelerate the progression of liver diseases to NAFLD^7–9^. Hepatic steatosis is characterized by dysfunctional hepatic fatty acid oxidation, excessive ectopic lipid accumulation, and intracellular redox imbalance. In recent years, the effect of BPA on hepatic fat metabolism has been studied, and its mechanisms mainly focus on oxidative stress, inflammation, and mitochondrial dysfunction^10–12^.

The endoplasmic reticulum (ER), one of the important organelles, participates in protein synthesis, folding, packing, and transport, as well as calcium ion storage via several chaperones and glycosidases^13,14^. The ER in hepatocytes is remarkably adaptable to intracellular and extracellular alterations, preserving critical hepatic metabolic processes. However, various stresses such as hyperlipidemia, inflammation, viruses, and medications, disrupt ER homeostasis in hepatocytes, promoting ER stress (ERS) and leading to hepatic lipid metabolism disorder and liver diseases^14–16^. ER stress is a self-protective mechanism of cells under stress, which causes unfolded and misfolded protein aggregation in the ER lumen, disrupts the calcium balance and triggers the unfolded protein response. The restoration of normal functions is facilitated by ER overload response and apoptotic signaling pathways, such as the activating-transcription factor 6 pathway, protein kinase RNA-like ER kinase pathway, and inositol-requiring enzyme 1 pathway^17^. BPA treatment promotes abnormal hepatic lipid metabolism via ER stress in ovariectomized female mice consuming a high-fat diet^18^. However, whether ER stress plays a role in the pathogenesis of hepatic lipid metabolic disorder induced by BPA alone is unclear.

Sulforaphane (SFN) is one of the most extensively investigated natural isothiocyanates which plays a role in improving lipid synthesis and catabolism^19–21^. One of the main target organelles of SFN is the ER. SFN stimulates the production of proteins associated with ER-stress response in addition to the activation of proteins related to antioxidant response^22^. SFN can reduce ER stress and reactive oxygen species (ROS) by increasing thioredoxin-1 and NADPH quinone oxidoreductase-l levels^22^. Although SFN can improve high-fat-diet-induced NAFLD^23^, no studies are available regarding the effect of SFN on BPA-induced liver lipid metabolism. Therefore, we hypothesized that BPA could activate ER stress, which would lead to hepatic lipid accumulation, and that SFN could alleviate these BPA effect by inhibiting ER stress.

## Methods

### Cell culture

Human immortalized liver LO2 cells were obtained from the Chinese Academy of Sciences’ Shanghai Institute of Cell Biology (Shanghai, China). The cells were incubated in RPMI-1640 medium (Gibco, Grand Island, NY, United States) under 5% CO_2_ at 37 °C with 10% FBS (Gibco), 0.1 mg/mL streptomycin, and 100 IU/mL penicillin (Gibco). 4-Phenylbutyric acid (4-PBA) was obtained from MedChem Express (New Jersey, United States). BPA and sulforaphane were obtained from Sigma-Aldrich (St. Louis, MO, USA). BPA, sulforaphane, and 4-PBA were dissolved in DMSO, while the control cells received the same amount of DMSO (0.1%). Then, LO2 cells were exposed to BPA and/or sulforaphane and/or 4-PBA for 24 h. These cells were then pretreated with 4-PBA (0.5 mM) for 2 h, followed by treatment with BPA.

### Antibodies and chemicals

Antibodies to the phosphorylation of the CCAAT-enhancer-binding protein homologous protein (CHOP), spliced X-box binding protein 1 (XBP1s), and α-subunit of eIF2 (p-eIF2α) were purchased from Affinity Biosciences (Victoria, AU, USA). GAPDH antibody was purchased from Bioworld (Shanghai, China). Anti-rabbit secondary antibody was provided by Signaling Technology (Danvers, MA, USA). All antibodies were used at the manufacturer’s recommended dilutions.

### Cell viability assay

In 96-well plates containing 100 μL medium and 1 × 10^4^ LO2 cells/well, different dosages of BPA and/(or) sulforaphane were added and incubated for 24 h. Cell viability following the treatment was assessed based on the results of the MTT assay. The methylthiazoletetrazolium test solution (5 mg/mL) was added to each well and the plate was incubated at 37 °C for 4 h. After removing the MTT-containing media, the precipitants were dissolved in DMSO. A microplate reader (TECAN, Austria) was used to measure the absorbance at 490 nm. All measurements were repeated thrice.

### BODIPY 493/503 and image acquisition

The lipid droplets (LD) dynamics in LO2 cells were assessed with BODIPY 493/503. After treatment, LO2 cells were rinsed thrice with PBS before fixing with 4% paraformaldehyde for 20 min at room temperature. The cells were then treated in BODIPY 493/503 (2 μM) at 37 °C for 30 min. 4′,6-Diamidino-2-phenylindole (DAPI) was incubated at room temperature for 20 min for nuclear staining. The images were captured with a 488-nm argon-ion laser. Confocal microscopy was performed with the Zeiss confocal laser scanning microscope 700 (Zeiss LSM 700, Germany), and photographs were processed with the ImageJ software (National Institutes of Health, Bethesda, MD).

### Determination of triglyceride (TG) and cholesterol contents

The amounts of TG and cholesterol in LO2 cells were measured by using the TG assay kits and total cholesterol (TC) assay kits (Jiancheng Bioengineering, China). According to the manufacturer’s instructions, the values of TG and TC were analysed and normalized using the total amount of protein. Each experiment was repeated at least thrice.

### Animals experiment

Male C57/BL6J mice (age: 4 weeks; weight: 18–22 g) were obtained from the Animal Research Center of Nanjing Medical University (Nanjing, Jiangsu, China). The mice were housed in a specific pathogen-free facility at Nanjing Medical University, where they were kept under 12-h light/dark cycles at a constant temperature of 22–26 °C. The animals were provided free access to feed and drink. The research protocol was approved by the Animal Care and Welfare Committee of Nanjing Medical University (IACUC-1903007). All experiments were performed in compliance with the relevant regulations and guidelines of the institution. This study was reported in accordance with the ARRIVE guidelines^24^.

After a 2-week adaptation period, the mice were randomly assigned to 3 groups (n = 6): control, BPA group (BPA), and sulforaphane intervention group (BPA + SFN). The mice in the BPA treatment group received subcutaneous injections of BPA (100 μg/kg body weight, dissolved in ethanol, and then diluted in maize oil). To examine the effect of BPA in vivo, previous studies have used different methods including gavage, oral feed, drinking water, and subcutaneous administration^25–30^. In a previous study, administration of BPA (100 μg/kg /day) by the route of subcutaneous injection for 15 days has been shown to induce hyperinsulinemia and insulin resistance in adult male C57BL/6J mice^28^. In another study, subcutaneous injection of BPA at 100 μg/kg/day for four weeks resulted in impaired glucose metabolism in mice^29^. Therefore, in the present study, BPA was injected subcutaneously into adult male C57BL/6J mice at a dose of 100 μg /kg/day.

Sulforaphane dissolved in ethanol and diluted in PBS was intraperitoneally administered to the mice of the SFN treatment group (10 mg/kg body weight), along with 100 μg/kg body weight of BPA. To examine the in vivo effects of SFN, previous studies have employed different delivery methods, including oral, intravenous, subcutaneous and intraperitoneal^31–35^. In one study, delivery of SFN via intraperitoneal injection (10 mg/kg/day) for 4 weeks has been shown to ameliorate elevated liver triglyceride levels in C57/BL6J mice induced by high-fat diet^34^. Therefore, in the present study, SFN was injected intraperitoneally at a dose of 10 mg/kg/day into C57BL/6J mice. The mice in the control group received injections of PBS and ethanol-dissolved maize oil in the same amounts. BPA and SFN were administered to the mice for a total of 6 weeks. The body weight of mice in each group was measured every week. The mice were euthanized after the last BPA dose treatment , and the liver tissues were collected for further examination.

### Histological analysis

The liver tissues were embedded in paraffin after treatment with a 10% formaldehyde solution overnight. H&E staining of the tissue slides was performed for the examination of the tissue morphology. The Panoramic Scanner (3DHISTECH, Budapest, Hungary) was used to analyze the liver slices.

### RNA isolation and quantitative real-time PCR (qRT-PCR)

TRIzol reagent (Invitrogen, Carlsbad, CA, USA) was used to extract the total RNA from the cells and liver tissues. The mRNA levels of peroxisome proliferator-activated receptor gamma (PPARγ), sterol regulatory element-binding transcription factor 1 (SREBF1), fatty Acid Synthase (FASN) and diacylglycerol-O-acyltransferase-1 (DGAT-1) were determined by qRT-PCR. Polymerase chain reaction primers used in this study are listed in Table 1.

**Table 1:** Primer sequences used for real-time quantitative PCR.

Species	Gene	Forward primer	Reverse primer
Human	PPARγ	5′-TTCAGGGCTGCCAGTTTCG-3′	5′-ATTTGAGGAGAGTTACTTGGTCGTT-3′
Human	SREBF1	5′-AGCCCCTCAGATACCACCAGC-3′	5′-CACCAAGGAGACGAGCACCAAC-3′
Human	FASN	5′-GTGTCCACCAGCAACATCAGC-3′	5′-TCTCCAGCAAGCCATCTCTCAA-3′
Human	DGAT-1	5′-TGAGCGTCCCTCTGCGAA-3′	5′-GCCATAGTTGCCCTGGAAAAAG-3′
Human	GAPDH	5′-CAAGGTCACCATGACAACTTTG-3′	5′-GTCCACCACCCTGTTGCTGTAG-3′
Mice	PPARγ	5′-GTGCCAGTTTCGATCCGTAGA-3′	5′-GGCCAGCATCGTGTAGATGA-3′
Mice	SREBF1	5′-TGCGTGGTTTCCAACATGAC-3′	5′-TGGCCTCATGTAGGAATACCCT-3′
Mice	FASN	5′-GGAGGTGGTGATAGCCGGTAT-3′	5′-TGGGTAATCCATAGAGCCCAG-3′
Mice	GAPDH	5'-TGTGTCCGTCGTGGATCTGA-3'	5'-TTGCTGTTGAAGTCGCAGGAG-3'

### Western blotting

After the appropriate treatments, the liver tissues and cells were collected and lysed in RIPA buffer containing 1X protease inhibitor cocktail (Pierce) and EDTA. The protein content of the total protein lysates was determined by the Bradford Protein Assay Kit. On a 10% SDS-PAGE, equal amounts of 60 μg of proteins were separated and transferred to PVDF membranes (Bio-Rad). After blocking with 5% nonfat dry milk, the membranes were incubated with suitable primary antibodies (1:500–1:1000 dilution) at 4 °C on a rotating shaker overnight. After flushing in Tris-buffered saline/Tween (TBS/T), the membranes were incubated with horseradish peroxidase-conjugated secondary antibodies (1:10,000 dilution) at room temperature for 1 h. GAPDH was used as a loading control to quantitatively compare the expression of proteins. ImageJ software (National Institutes of Health, Bethesda, MD) was used to quantify the protein bands on blots for densitometric analyses.

### Statistical analysis

The mean and standard deviation of all experimental data were determined. Unpaired t-test and one-way ANOVA analyses were employed to examine the statistical differences between two or multiple groups. GraphPad Prism 7.0 software (USA, San Diego, CA) was used for statistical analysis and bar graph generation. *p* < 0.05 was considered to indicate statistical significance.

## Results

### BPA promotes lipid accumulation in LO2 cells

**Figure 1:**
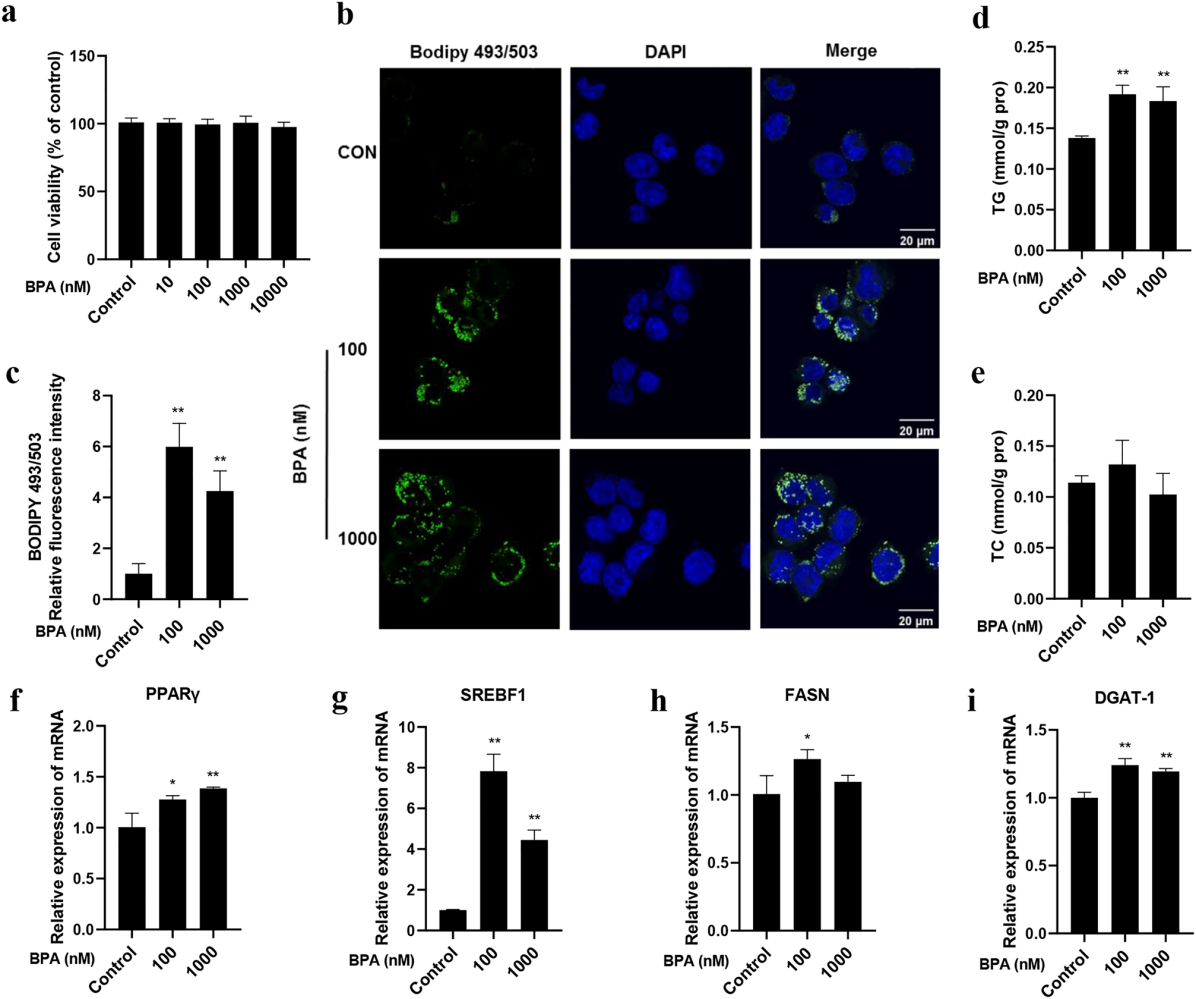
BPA promoted lipid accumulation in LO2 cells. (**a**) Different concentrations of BPA (10, 100, 1000, 10,000 nM) were added to LO2 cells for 24 h, and MTT assay was performed to assess the cell viability. (**b**) BODIPY 493/503 staining was performed for lipid droplets. Scale bar = 20 μm. (**c**) The relative quantification of BODIPY 493/503 fluorescence intensity. (**d**) TG levels and (**e**) cholesterol levels in LO2 cells. The mRNA expression levels of (**f**) PPARγ, (**g**) SREBF1, (h) FASN and (**i**) DGAT-1 were measured by qRT-PCR. Data were expressed as the mean ± SD of 3 independent experiments. *p < 0.05 and **p < 0.01, compared with control group.

The cytotoxic effect of BPA on LO2 cells was evaluated first. As shown in Fig. 1a, compared with the control cells, BPA treatment at the concentrations of 10,000 nM and less for 24 h did not affect the viability of LO2 cells considerably. Human exposure to BPA is widespread. However, according to the Chapel Hill BPA expert panel consensus statement, the concentration of unbound BPA in tissues and fluids is mainly in the range of 0.3–4.4 ng/mL (1.3–19.4 nM)^36^. A study reported that serum BPA concentrations in women with miscarriages were as high as 47.7 ng/mL (209.9 nM)^37^. In our previous study which investigated BPA-induced abnormalities in insulin signaling pathway and glucose metabolism in hepatocytes, we observed that the effect of BPA at 100 nM was the most significant among the three exposure concentrations of 1, 10, and 100 nM^38^. Therefore, based on above information, 100 and 1000 nM BPA concentrations were used in the subsequent experiments. As shown in Fig. 1b,c, BPA exposure promoted hepatic lipid accumulation, resulting in dramatic LDs (marked green with BODIPY493/503 staining) and higher TG levels (Fig. 1d). BPA treatment did not change TC levels considerably (Fig. 1e) (*p* > 0.05). We then evaluated the mRNA expression of the following lipogenesis-related genes in LO2 cells: PPARγ, SREBF1, FASN, and DGAT-1. It was shown that the mRNA expression levels of those genes were upregulated in BPA groups (Fig. 1f–i). These results suggest that BPA can induce lipid accumulation in human-immortalized liver LO2 cells.

### BPA induces hepatic lipid accumulation by activating ER stress in LO2 cells

**Figure 2:**
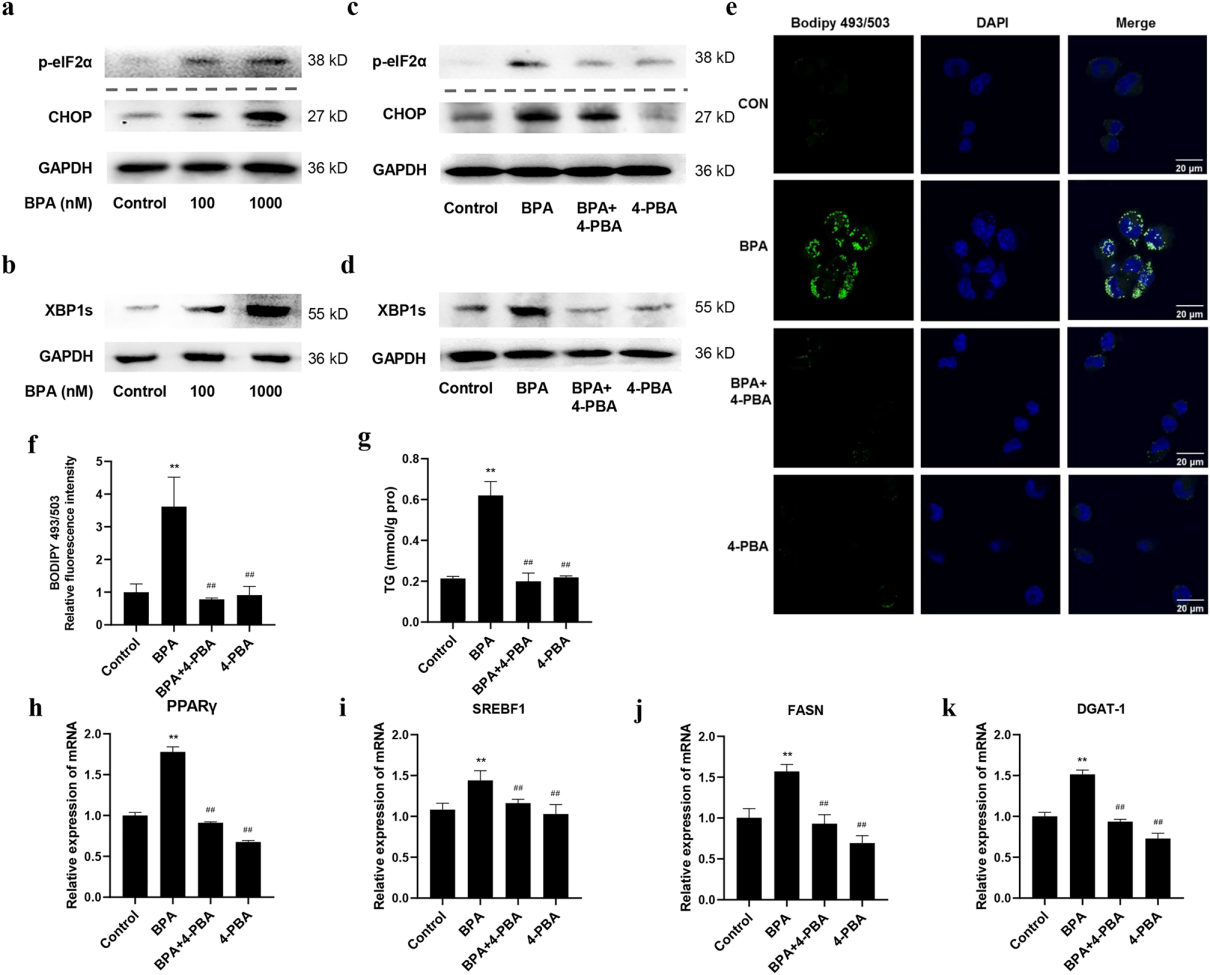
BPA-activated ER stress in LO2 cells. LO2 cells were treated with 100 nM and 1000 nM BPA for 24 h. The levels of (**a**) p-eIF2α, CHOP, and (**b**) XBP1s were measured by Western blotting. The blots of CHOP and GAPDH were cropped from different portions of the same membrane. The p-eIF2α blot was not cropped from the same membrane as the above two blots, but rather from the same sample. The blots of XBP1s and GAPDH were cropped from different portions of the same membrane. The original blots are presented in Supplementary Figs. S1, 2. LO2 cells were treated with 100 nM BPA and (or) 4-PBA for 24 h. The levels of (**c**) p-eIF2α, CHOP, and (**d**) XBP1s were measured by Western blotting. The original blots are presented in Supplementary Figs. S3, 4. (**e**) BODIPY 493/503 staining for lipid droplets. Scale bar = 20 μm. (**f**) Quantification indicating the relative BODIPY 493/503 fluorescence intensity. (**g**) The TG levels in LO2 cells. The mRNA expression levels of (**h**) PPARγ, (**i**) SREBF1, (**j**) FASN, and (**k**) DGAT-1 were measured by qRT-PCR. Data are expressed as the mean ± SD of 3 independent experiments. *p < 0.05 and **p < 0.01, compared with control group; #*p* < 0.05 and ##*p* < 0.01, compared with BPA group.

After treatment with BPA for 24 h, the levels of ER stress pathway proteins, including p-eIF2α, CHOP, and XBP1s, were determined. As expected, BPA treatment considerably increased p-eIF2α, CHOP, and XBP1s levels (Fig. 2a,b). These data suggested that BPA impeded ER homeostasis. 4-PBA is an ER stress inhibitor with low-molecular-weight that can stabilize the conformation of proteins, improve the folding capacity of the ER, and facilitate the trafficking of mutant proteins to suppress ER stress^39^**.** In this study, 4-PBA was used to inhibit ER stress signaling and to examine the role of ER stress in BPA-induced lipid accumulation in LO2 cells. The western blotting results showed that the levels of p-eIF2α, CHOP, and XBP1s were reduced in cells pre-treated with 4-PBA, suggesting that 4-PBA inhibited ER stress (Fig. 2c,d). Furthermore, 4-PBA prevented BPA-induced upregulation of p-eIF2α, CHOP, and XBP1s (Fig. 2c,d). Meanwhile, BPA-triggered hepatic lipid accumulation was diminished in 4-PBA-pretreated cells (Fig. 2e,f). Similar results were also observed for TG levels (Fig. 2g) and the mRNA levels of the lipogenesis-related genes (Fig. 2h–k). Collectively, these data suggest that ER stress plays a regulatory role in lipid metabolism disorder induced by BPA.

### SFN ameliorates BPA-induced lipid accumulation and ER stress in LO2 cells

**Figure 3:**
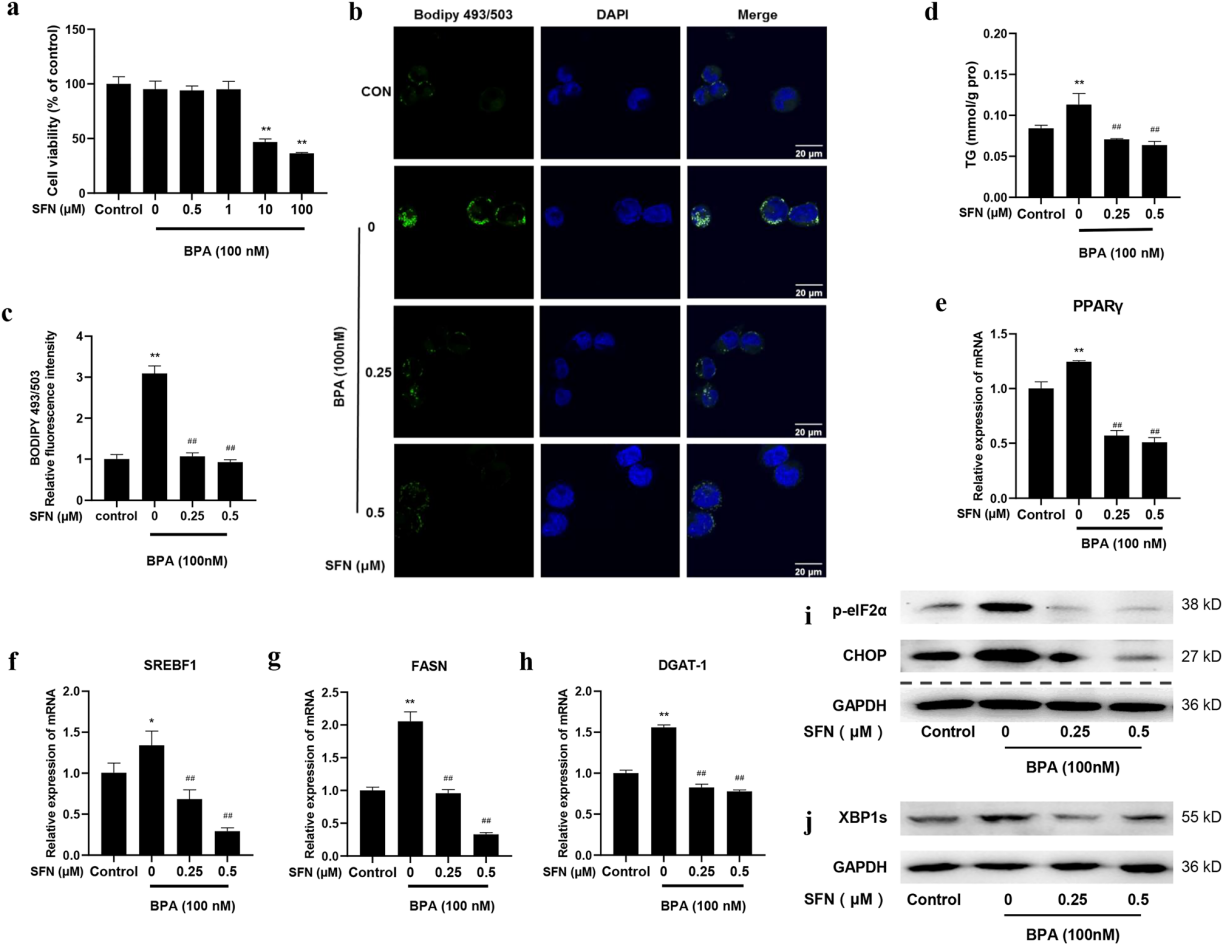
SFN ameliorated BPA-induced lipid accumulation and ER stress in LO2 cells. (**a**) LO2 cells were treated with 100 nM BPA and designated concentrations of SFN (0, 0.5, 1, 10, and 100 μM) for 24 h. Cell viability was evaluated by MTT assay. (**b**) BODIPY 493/503 staining for lipid droplets. Scale bar = 20 μm. (**c**) Quantification of the relative BODIPY 493/503 fluorescence intensity. (**d**) The TG levels in LO2 cells. The mRNA expression levels of (**e**) PPARγ, (**f**) SREBF1, (**g**) FASN, and (**h**) DGAT-1 were detected by qRT-PCR. The levels of (**i**) p-eIF2α, CHOP, and (**j**) XBP1s were measured by Western blotting. The blots of p-eIF2α and GAPDH were cropped from different portions of the same membrane. CHOP blot was not cropped from the same membrane as the above two blots but from the same sample. The blots of XBP1s and GAPDH were cropped from different parts of the same membrane. The original blots are presented in Supplementary Figs. S5, 6. Data are expressed as the mean ± SD of 3 independent experiments. *p < 0.05 and **p < 0.01, when compared with the control group; #*p* < 0.05 and ##*p* < 0.01, when compared with the BPA group.

As shown in Fig. 3a, SFN (0–1 μM) did not exhibit obvious cytotoxicity in LO2 cells when combined with 100 nM BPA. Therefore, we investigated the effects of 0.25 μM and 0.5 μM SFN on BPA-treated LO2 cells. The results showed SFN treatment effectively prevented the increase in hepatic lipid accumulation and TG levels triggered by BPA in LO2 cells (*p* < 0.01) (Fig. 3b–d). Simultaneously, SFN down-regulated the mRNA expression of BPA-induced lipogenesis-related genes and the levels of ER stress markers (Fig. 3e–j). These results suggest that SFN improves abnormal lipid metabolism and ER stress caused by BPA in LO2 cells.

### SFN attenuates BPA-induced hepatic lipid accumulation and ER stress in mice

**Figure 4:**
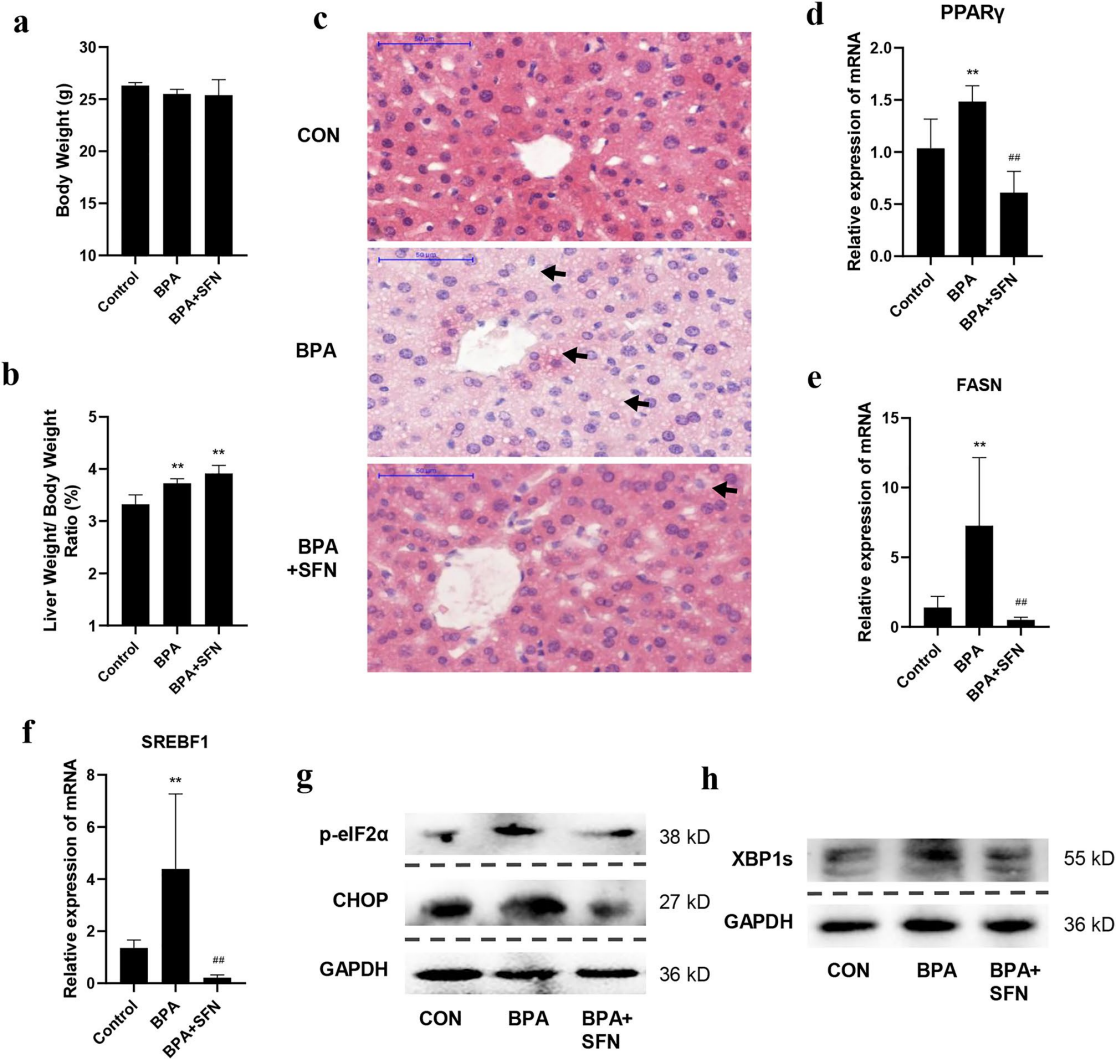
SFN attenuated BPA-induced lipid accumulation and ER stress in mice. C57/BL6J mice were randomly divided into three groups: control, BPA, and SFN intervention group. Mice in BPA group were injected subcutaneously with 100 μg/kg of BPA; mice in SFN group were injected with the same dose of BPA subcutaneously, while 10 mg/kg of SFN were injected intraperitoneally; mice in the control group were injected subcutaneously and intraperitoneally with the same amount of diluted ethanol and corn oil. (**a**) The body weight of each group of mice was weighed. (**b**) The liver of each group of mice was weighed, and the ratio of liver/body weight was calculated. (**c**) H&E of the liver tissues in the experimental mice. The mRNA levels of (**d**) PPARγ, (**e**) FASN, and (**f**) SREBF1 in mice were detected by qRT-PCR. The expression of (**g**) p-eIF2α, CHOP, and (**h**) XBP1s was measured by Western blotting. The blots of p-eIF2α, CHOP, and GAPDH were not cropped from the same membrane, but rather from the same sample. The blots of XBP1s and GAPDH were not cropped from the same membrane, but rather from the same sample. The original blots are presented in Supplementary Figs. 7, 8. Data are expressed as the means ± SD of 6 mice per group. *p < 0.05 and **p < 0.01, when compared with the control group; #*p* < 0.05 and ##*p* < 0.01, when compared with the BPA group.

We then verified our in vitro experiments’ results in vivo by randomly dividing C57/BL6J mice into three groups according to body weight as follows: control, BPA, and SFN-intervention groups. After treatment, the body weights and liver weights of mice were measured. No considerable differences were observed in body weights among the three groups (Fig. 4a). BPA considerably increased the liver/body weight ratio (Fig. 4b) (*p* < 0.01). We further investigated whether BPA treatment promoted hepatic lipogenesis in these mice. H&E staining showed abnormal LDs in BPA-treated mouse liver tissues, which was attenuated by SFN treatment (Fig. 4c). Furthermore, we detected the expression of lipogenesis-related genes in mouse livers. As shown in Fig. 4d–f, BPA upregulated lipogenesis-related gene expression, whereas SFN downregulated their expression. In terms of the ER stress pathway, it was revealed that BPA treatment elevated ER stress marker’s levels in mouse livers (Fig. 4g,h), and SFN suppressed the activation of ER stress pathway. These results suggest that BPA exposure promotes ER stress and lipid accumulation in the mouse liver, and these effects were diminished by SFN.

## Discussion

The present study illustrated that BPA promoted lipid accumulation and upregulated the expression of lipogenesis-related genes in human hepatocyte LO2 cells and mouse livers by activating the ER stress pathway, which was ameliorated by blocking ER stress or SFN treatment.

The liver is the primary target organ of BPA. It is also the most important organ for lipid synthesis and plays a key role in maintaining lipid metabolic homeostasis. Previous studies in animals and humans have shown that exposure to BPA may increase the accumulation of lipids in hepatocytes and the levels of key enzymes in lipid synthesis^40,41^. However, the molecular mechanisms by which BPA causes metabolic abnormalities in hepatic lipids remain to be elucidated. In the present study, we also found that BPA increased hepatic accumulation of lipids, TG levels, and the mRNA levels of lipogenesis-related genes. However, we did not observe differences in cholesterol levels in LO2 cells treated with BPA. In contrast, a study reported by Li et al. showed that hepatic cholesterol content was increased when C57BL/6 mice and HepG2 cells exposed to BPA^42,43^. This suggests that the effects of BPA on specific lipids may differ, which might be related to factors such as patients, BPA doses, and treatment duration, etc.

SREBF1 is a membrane-bound transcription factor that activates the expression of most genes required for hepatic lipogeneses, such as ACC, FAS, and SCD1^44,45^. PPARγ is an additional reinforcing lipogenic signal that assists sterol regulatory element-binding protein-1c in triggering hepatic steatosis development^46^. PPARγ is also involved in regulating DGAT-1, which catalyzes the final step in triacylglycerol synthesis^47^. We speculated that BPA might regulate lipid metabolism by affecting the SREBF1 and PPARγ pathway. In the present study we found that the mRNA levels of SREBF1, PPARγ, FASN and DGAT-1 in LO2 cells and mice liver tissues treated with BPA were markedly increased. These results suggest the mechanism by which BPA increased lipid synthesis may be related to the activation of SREBF1 and PPARγ pathway.

A literature search revealed that the mechanistic studies of BPA-induced liver damage mainly focused on mitochondrial dysfunction, ROS formation, and inflammatory states^48–50^, while relative less studies were available regarding ER stress. The ER, an intracellular membranous network structure that integrates cellular signaling and homeostasis, is also the primary location for lipid production in hepatocytes^14^. Some conditions, such as glucose and energy deprivation, high cholesterol, and protein glycosylation inhibition, can activate ER stress^51–53^. ER stress can cause de novo lipogenesis, directly affecting hepatic lipid metabolism^14^. Upon ER stress, ER membranes is cleaved and the released SREBF enter the nucleus, activating the promoter of sterol biosynthesis, thus increasing fatty acid, triglycerides and cholesterol synthesis^54,55^. Therefore, we hypothesized that BPA induced hepatic lipogenesis by activating the ER stress pathway to enhance SREBF signaling. In LO2 cells treated with BPA, we found that BPA activated the ER stress pathway by increasing p-eIF2α, CHOP, and XBP1s levels; however, BPA-induced lipid metabolism abnormality was reversed by ER stress inhibitor 4-PBA, suggesting the important role of ER stress in BPA-triggered hepatic lipid abnormality. In the animal experiment, the levels of ER stress markers in the liver of BPA-treated mice were also increased considerably. Previous studies showed that BPA treatment induced lipid accumulation in the liver and enhanced ER stress in ovariectomized mice fed on a high-fat diet, and in Watanabe heritable hyperlipidemic rabbits^18,56^. Nevertheless, the present results illustrated for the first time that BPA treatment alone affected liver fat metabolism and ER stress in vivo and in vitro. These results suggest that low-dose BPA may interfere with hepatic lipid metabolism and active ER stress, even if it does not interact with factors such as a high-fat diet.

Recent studies have shown that SFN can improve lipid accumulation and the levels of key lipogenic enzymes in hepatocytes and liver tissues induced by high-fat diet, through the mechanisms mainly involving oxidative stress, inflammation, and insulin resistance^57–59^. One study showed that SFN inhibited the expression of ACC1 and SCD1 by targeting the ERS response pathway, thereby improving high-fat-diet-induced abnormal hepatic lipid metabolism^60^. Modulation of the ER stress response pathway via the nuclear factor erythroid 2-related factor 2-regulated antioxidant pathway contributed to the action of SFN^22^. In the present study we hypothesized that SFN improved BPA-induced lipid metabolism by inhibiting the ER stress pathway. We depicted that SFN ameliorated the accumulation of lipids in hepatocytes and the levels of key lipogenic enzymes through inhibition of the ERS pathway. In in vivo experiment, however, SFN treatment did not resulted in considerable decrease in the liver/body weight ratio compared with BPA group, which might be related to the relative short intervention duration of SFN. Nevertheless, the present study is the first investigation to reveal the ameliorative effect of SFN on BPA-induced lipid metabolic abnormalities and ER stress in both in vitro and in vivo settings. In conclusion, our findings suggested that BPA activated ER stress to promote hepatic lipid accumulation, and that SFN diminished BPA-triggered effects on lipid metabolic abnormalities by alleviating ER stress.

## Supplementary Information

Supplementary Figures.

## Data Availability

The datasets used and/or analyzed during the current study are available from the corresponding author on reasonable request.
