# Supplementary material for: Sulforaphane ameliorates bisphenol A-induced hepatic lipid accumulation by inhibiting endoplasmic reticulum stress
Source: Sci Rep. 2023 Jan 20;13:1147. doi: 10.1038/s41598-023-28395-5 (PMC9859828; doi:10.1038/s41598-023-28395-5)
Supplement: Supplementary file 1 — Supplementary Figures. [file 41598_2023_28395_MOESM1_ESM.pdf]

Supplemental information

Sulforaphane Ameliorates Bisphenol A-Induced Hepatic Lipid Accumulation by Inhibiting Endoplasmic Reticulum Stress

Lixia Hong[1], Yide Xu[1], Dongdong Wang[1], Qi Zhang[1], Xiaoting Li[1], Chunfeng Xie[1], Jieshu Wu[1], Caiyun Zhong[1*], Jinyan Fu[2*], Shanshan Geng[1*]

The complete membranes of XBP1s and GAPDH from one of our experiments including various exposure times were shown below:

XBP1s

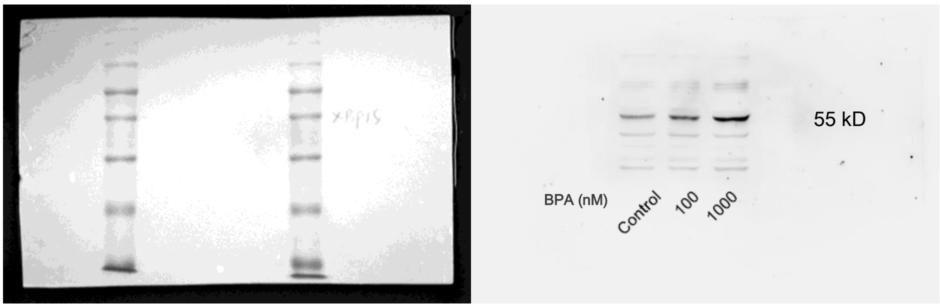

exposure time: 3 seconds

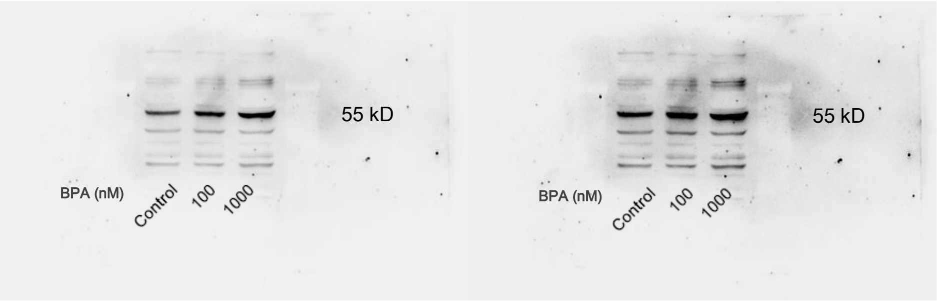

exposure time: 10 seconds

exposure time: 30 seconds

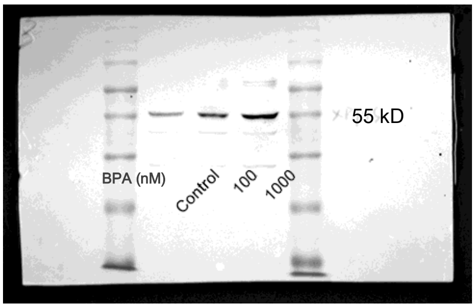

GAPDH

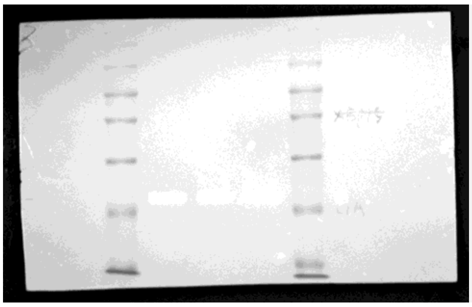

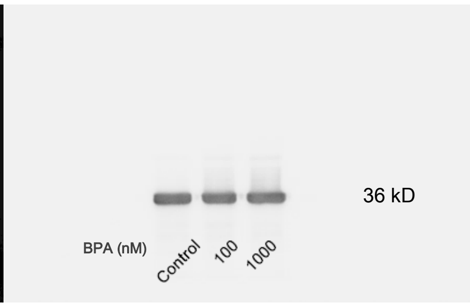

exposure time: 0.05 seconds

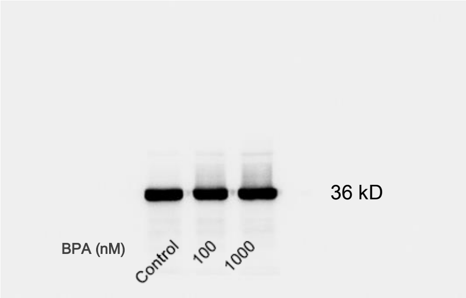

exposure time: 0.5 seconds

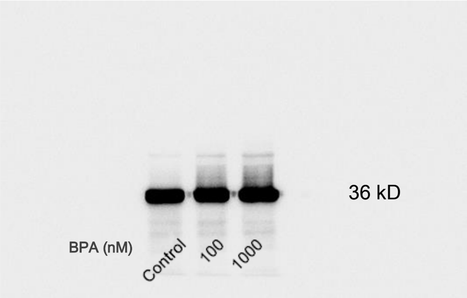

exposure time: 10 seconds

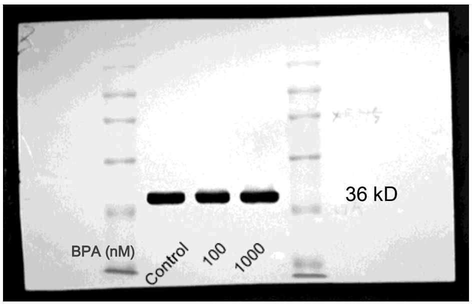

Fig.S1

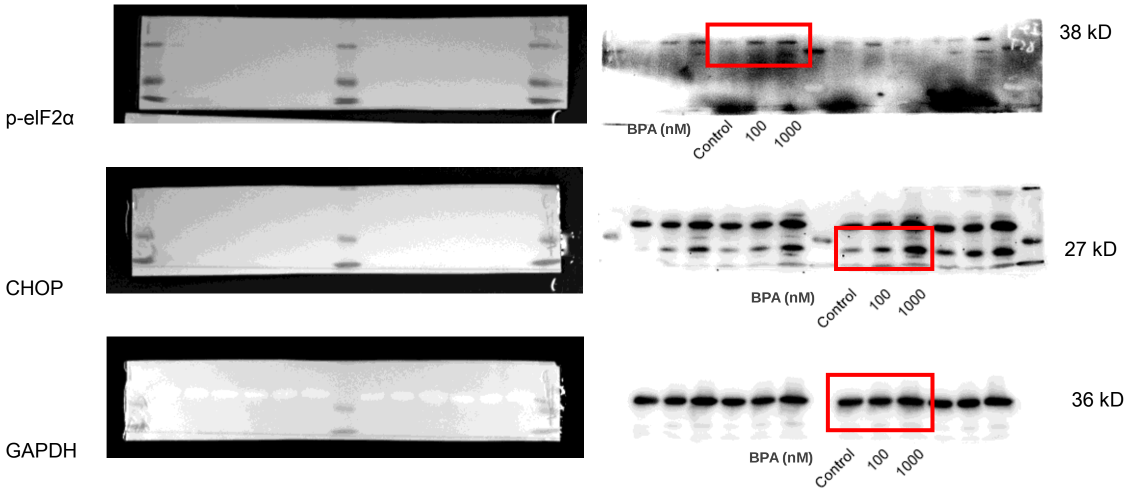

The blots of GAPDH including various exposure times were shown below:

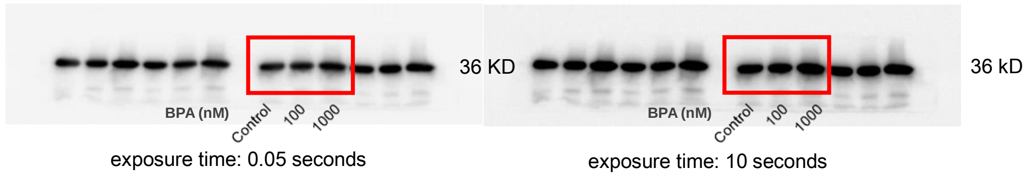

Fig.S2

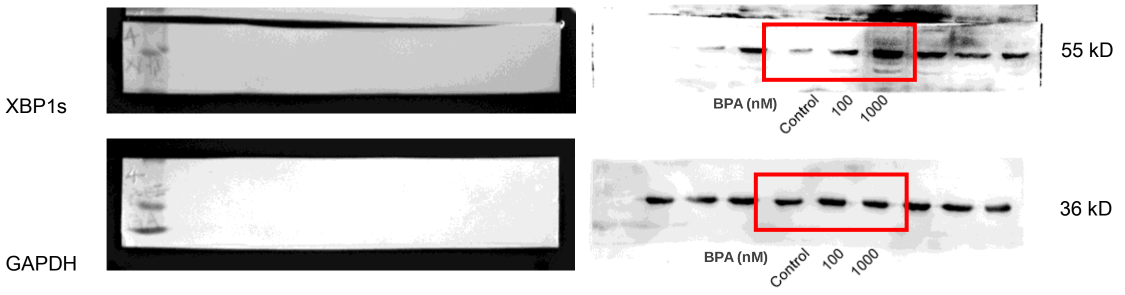

Fig.S3

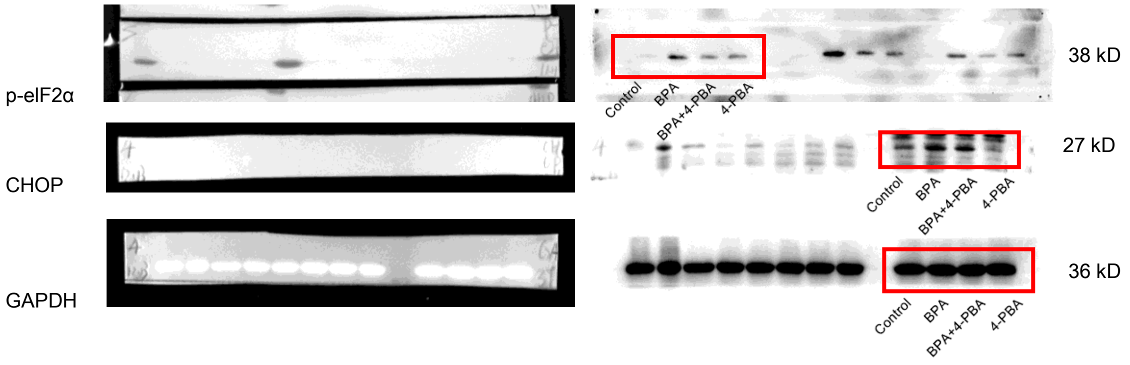

Fig.S4

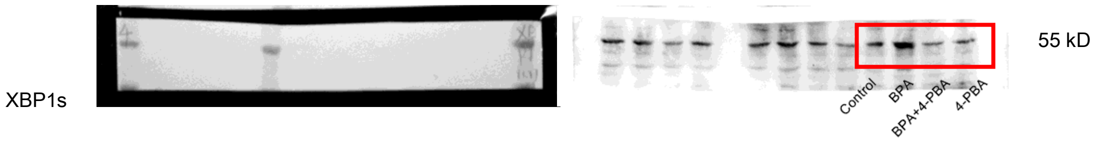

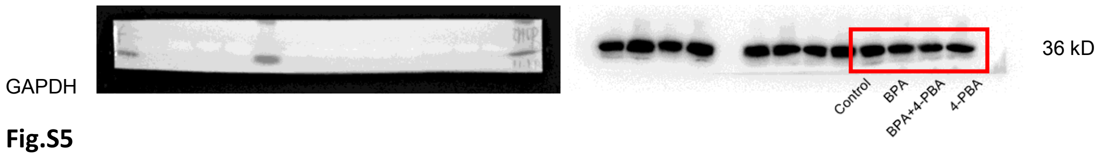

Fig.S5

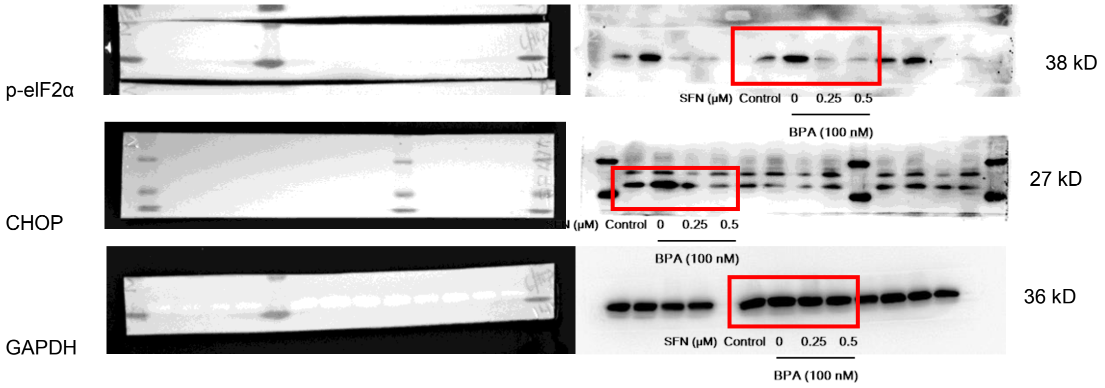

Fig.S6

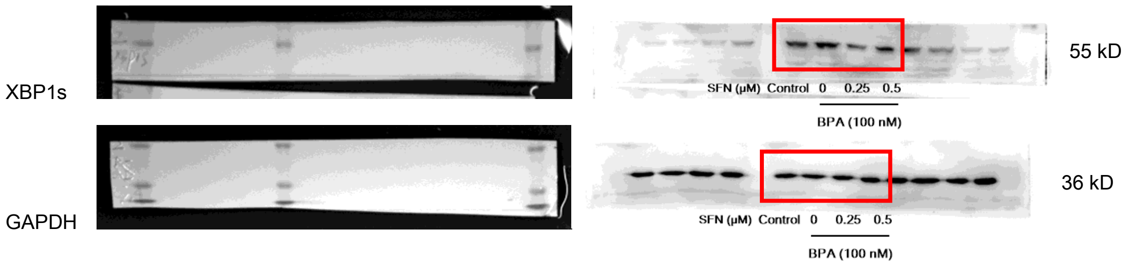

Fig.S7

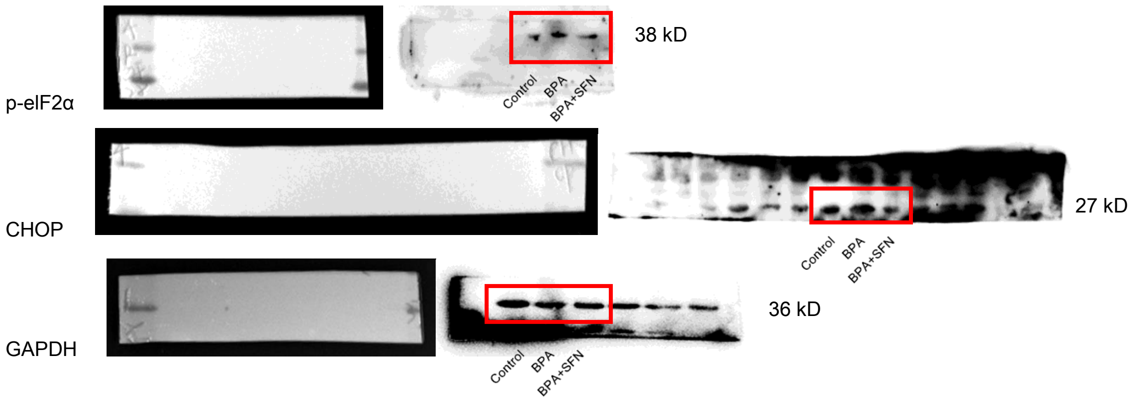

Fig.S8

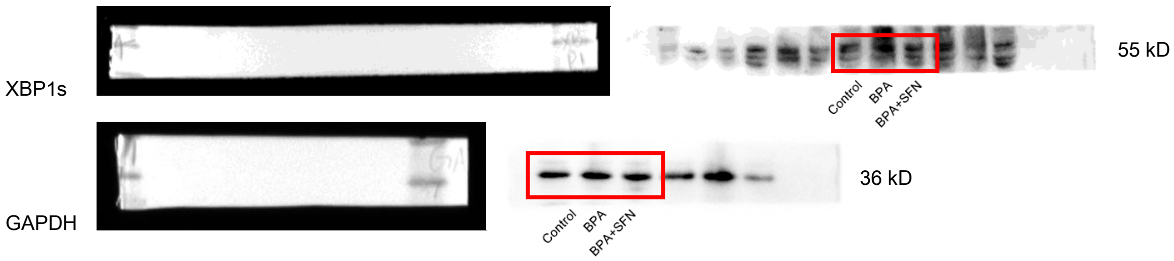

The images processing follows the guidelines of the digital image and integrity policies.

Brightness and contrast adjustment was applied equally across the entire images and the blots,

presented in the manuscript text, were cropped using Adobe Photoshop software (Copyright © 2020 Adobe).
